# Genetic architecture and QTL selection response for Kernza perennial grain domestication traits

**DOI:** 10.1007/s00122-022-04148-2

**Published:** 2022-06-28

**Authors:** Jared Crain, Steve Larson, Kevin Dorn, Lee DeHaan, Jesse Poland

**Affiliations:** 1 Department of Plant Pathology, Kansas State University, 4024 Throckmorton Plant Sciences Center, Manhattan, KS 66506 USA; 2 USDA-ARS, Forage and Range Research, Utah State University, Logan, UT 84322 USA; 3 USDA-ARS, Soil Management and Sugarbeet Research, Fort Collins, CO 80526 USA; 4 The Land Institute, 2440 E. Water Well Rd, Salina, KS 67401 USA; 5 Center for Desert Agriculture, King Abdullah University of Science and Technology, Thuwal, Saudi Arabia

## Abstract

**Key message:**

Analysis of multi-year breeding program data revealed that the genetic architecture of an intermediate wheatgrass population was highly polygenic for both domestication and agronomic traits, supporting the use of genomic selection for new crop domestication.

**Abstract:**

Perennial grains have the potential to provide food for humans and decrease the negative impacts of annual agriculture. Intermediate wheatgrass (IWG, *Thinopyrum intermedium*, Kernza®) is a promising perennial grain candidate that The Land Institute has been breeding since 2003. We evaluated four consecutive breeding cycles of IWG from 2016 to 2020 with each cycle containing approximately 1100 unique genets. Using genotyping-by-sequencing markers, quantitative trait loci (QTL) were mapped for 34 different traits using genome-wide association analysis. Combining data across cycles and years, we found 93 marker-trait associations for 16 different traits, with each association explaining 0.8–5.2% of the observed phenotypic variance. Across the four cycles, only three QTL showed an *F*_*ST*_ differentiation > 0.15 with two corresponding to a decrease in floret shattering. Additionally, one marker associated with brittle rachis was 216 bp from an ortholog of the *btr2* gene. Power analysis and quantitative genetic theory were used to estimate the effective number of QTL, which ranged from a minimum of 33 up to 558 QTL for individual traits. This study suggests that key agronomic and domestication traits are under polygenic control and that molecular methods like genomic selection are needed to accelerate domestication and improvement of this new crop.

**Supplementary Information:**

The online version contains supplementary material available at 10.1007/s00122-022-04148-2.

## Introduction

Perennial grain crops have the potential to revolutionize agriculture. In contrast to their annual counterparts that require regular tillage and anthropogenic disturbances (Crews et al. 2018), perennials could provide a host of ecosystem services (Glover et al. 2010; Crews et al. 2018). Documented ecosystem services by perennial crops include reduced nitrate leaching (Culman et al. 2013; Jungers et al. 2019), more complex soil communities (Culman et al. 2010), greater ability to store and retain carbon (Sprunger et al. 2018), and increased nutrient cycling (Pugliese et al. 2019). Although there are currently no-large scale perennial grain crops, the development and utilization of such crops could transform both the sustainability and economic foundations of agriculture (Crews et al. 2018).

Intermediate wheatgrass (IWG, *Thinopyrum intermedium* (Host) Barkworth and D.R. Dewey, trade name Kernza) is a close perennial relative of wheat and has a similar allohexaploid genome (2*n* = 6*x* = 42) with an estimated genome size of 12.75 Gb (Vogel et al. 1999). Based on comparisons of nearly 100 species of perennial grasses, IWG was first identified for domestication in the 1980s by work at the Rodale Institute (Kutztown, Pennsylvania, USA) because of its relatively large seed size, promising yield, and palatability (Wagoner 1990a, b). In addition to more favorable agronomic traits, the grain has a soft endosperm comparable to soft wheat (*Triticum aestivum*) (Bajgain et al. 2020b), with quality evaluations showing IWG has higher levels of amino acids, protein, and bran percentage than wheat (Becker et al. 1991). Even though IWG has higher grain yield than many perennials, its yield is estimated to only be 10–20% of annual wheat (DeHaan et al. 2014; DeHaan and Ismail 2017), necessitating sustained breeding efforts to increase the yield of this potential grain crop. Additionally, several other agronomic and domestication traits such as reduced shattering, increased seed size, and improved threshability are needed to make IWG a commercial crop.

Uninterrupted breeding efforts to improve IWG have been conducted at The Land Institute (TLI), Salina, Kansas, USA, since 2003 (DeHaan et al. 2018), with new breeding programs being initiated in Minnesota, USA (2011), Manitoba, Canada (2011), Utah, USA (2019), and Uppsala, Sweden (2019) (Cattani 2016; Zhang et al. 2016; Bajgain et al. 2020b). While the initial cycles of selection relied on recurrent phenotypic selection (Zhang et al. 2016; DeHaan et al. 2018), advances in low cost, high-throughput DNA sequencing have permitted IWG breeding to harness the power of genomic selection (GS) (Zhang et al. 2016; Bajgain et al. 2020a; Crain et al. 2020a, 2021a, b). Within TLI’s breeding program, GS has reduced the breeding cycle from three years to one year per cycle (DeHaan et al. 2018) and simultaneously maintained an estimated 8% year^−1^ increase in spike yield (Crain et al. 2021a). Furthermore, decreased sequencing cost has resulted in a wealth of genomic information for crop improvement including genetic maps (Kantarski et al. 2016) and a draft genome sequence (https://phytozome-next.jgi.doe.gov/info/Tintermedium_v2_1). These genomic resources have enabled genome-wide association studies (GWAS) for agronomic traits including seed size (Zhang et al. 2017; Larson et al. 2019), flowering time (Altendorf et al. 2021c), and grain yield components (Bajgain et al. 2019; Larson et al. 2019) that can be used to better understand and guide IWG breeding.

Since initiating GS in 2017, TLI has completed four cycles of selection for reduced seed shattering, enhanced threshability to produce naked seed (free-threshing trait), increased seed mass, and higher spike yield. Even though selections have been primarily based on GS models for these few primary traits, up to 34 traits have been measured which allow for a holistic assessment of the breeding program. The estimated genetic gains have generally been favorable and at a more rapid rate than phenotypic selection alone, yet there has been some evidence of unanticipated results. Within the breeding program, increasing spike yield has been associated with increased seeds per spike, number of florets per spike, and florets per spikelet, yet the floret site utilization (FSU, referred to as percent seed set in Crain et al., 2021a) decreased, suggesting less efficient use of resources. While FSU has not been a direct target of selection in the TLI program, Altendorf et al. (2021a) have found that FSU was the primary driver of yield for spaced plants grown on 1 m centers (e.g. one-meter spacing between plants).

Within annual wheat, increasing the number of seeds per spikelet (Würschum et al. 2018) or spike fertility, percent of grain weight to total spike weight, has been shown to increase yield (Alonso et al. 2018), yet Philipp et al. (2018) reported that there appears to be little evidence that the number of spikelets per spike has been improved in elite varieties from landraces or wild germplasm. Although IWG is indeterminate for the number of fertile florets per spikelet and spikelets per spike, a key element that should be considered is the difference between annual and perennial life cycle, specifically whether a high yielding perennial grain crop is viable. Research has shown that perennials devote more resources below ground than do their annual counterparts and that this allocation is a precursor to switching between perennial and annual life cycles in natural ecosystems (Lindberg et al. 2020). Additionally, selecting for higher seed yield may induce concessions from below-ground resources and plant longevity (Vico et al. 2016).

While there are some arguments against perennial grains due to the hypothesized ecological and physiological limitation of perennial plants (Smaje 2015), current work suggests that favorable gains can be made through artificial selection (DeHaan et al. 2014; Zhang et al. 2016; Crain et al. 2021a). As breeding programs mature, they should assess whether the realized gains in perennial crops are matching the target gains for both agronomic yield and increased ecosystem services. Given the rapid cycling nature of the TLI IWG breeding program and the results from the first few cycles of GS (Crain et al. 2021a), our objectives are to (1) conduct a GWAS for observed traits to identify associated loci for key agronomic traits, (2) determine the genetic architecture of the observed traits, (3) assess allele frequency changes across the four cycles of selection for significant marker-trait associations, and (4) evaluate the potential selection opportunities to drive genetic gains for desirable physiological and agronomic outcomes such as high grain yield and high FSU.

## Materials and methods

### Plant material

All plant material used in this study came from the TLI breeding program, Cycles 6 to 9, with TLI-Cycle 6 being extensively described in DeHaan et al. (2018) and TLI-Cycles 7 to 9 detailed in Crain et al. (2021a, b). Briefly, TLI-Cycle 6 formed the initial training population for GS and consisted of 3,658 space-planted genets that were evaluated in 2016 and 2017 at Salina, KS (Crain et al. 2021b). As outcrossed IWG plants are all unique and heterozygous (excluding clones or ramets), the term “genet” herein refers to a genetically unique individual which is typically a single plant but possibly cloned ramets, while genotype herein refers to the DNA sequence of a particular genet (Zhang et al. 2016). Phenotypic data and pedigree-based relationships used to calculate predicted breeding values were used to select TLI-Cycle 6 genets that were randomly intermated to form TLI-Cycle 7. Genomic selection was used to identify 118 TLI-Cycle 7 genets, out of 4,183 genotyped, to intermate to form TLI-Cycle 8 seed. Another 1,216 TLI-Cycle 7 genets were selected for field evaluations to train future GS models and divided randomly between an irrigated and a non-irrigated site. Genets were space planted on 0.91 m centers in the fall of 2017 with phenotypic evaluations in 2018, 2019, and 2020. TLI-Cycle 8 and 9 were formed in a same manner with around 100 selected genets intermated to form each subsequent cycle out of nearly 3,500 genotyped genets. Planting was similar to TLI-Cycle 7, where individual genets were divided between irrigated and non-irrigated sites and planted on 0.91 m centers. The TLI-Cycle 8 training population consisted of 1,092 genets planted in the field in the fall of 2018 and evaluated during 2019 and 2020. The TLI-Cycle 9 training population was comprised of 1,004 genets, planted in the fall of 2019 with first-year phenotypic observations in 2020. Across all cycles, there was no replication of genets, thus each genet was evaluated as a unique single plant.

### Phenotypic assessment

Each year phenotypic traits were measured to evaluate genet performance, with a total of 34 unique traits (Crain et al. 2021a). Within the breeding program the most important traits which are key selection targets include shattering, percent free-threshing seed, seed mass, and spike yield. Shattering was rated on a scale of 0 to 5, where 0 indicated no shattering and 5 indicated more than 12 florets shattering per evaluated spike (DeHaan et al., 2018). From 2016 to 2018, shattering was considered a single trait; however, work by Altendorf (2021b) indicated that floret and brittle rachis shattering should be scored separately, so beginning in 2019 brittle rachis was scored as a separate trait in the IWG population. In addition, many other secondary traits including seeds spike^−1^, spikelets spike^−1^, florets spike^−1^, and FSU were evaluated. While most traits were assessed consistently across years and cycles, it should be noted that TLI-Cycle 6 had significant missing data due to flooding, and reduced data collection in 2020 reflected limited labor due to the COVID-19 pandemic. A subset of 1,470 TLI-Cycle 6 genets was selected to make approximately equal representation of genet number between cycles and follows previous work by Crain et al. (2021a).

A linear mixed model, Eq. 1, was used to calculate trait best linear unbiased predictors (BLUPs) for each genet using ASREML version 4.1 (Gilmour et al. 2015).

1 $$y = Xb + Zu + e$$

In Eq. 1, ***y*** is a vector of phenotypic observations, fixed and random effects are given by vector ***b*** and ***u*** respectively, and ***e*** is a vector of residuals. The incidence matrices ***X*** and ***Z*** allocate each fixed or random effect to their corresponding observation in **y** (Isik et al. 2017). For each model no fixed effects were added, so ***Xb*** reduces to the mean vector. Random effects that were normal, independent, and identically distributed ~ NIID(0, ${\sigma }_{effect}^{2}$) were included for site-year combination, multiple measurements for each genet representing observations across years, and a nugget effect for residual error variance. A random term for genet was included that had a mean 0 and a known variance–covariance matrix of the genomic relationship matrix (GRM) ~ (0, ${\sigma }_{genet}^{2}$ **GRM)**, which explained the genet effect that accounts for the relationship between genets using the GRM (Isik et al., 2017 pg 124–125). The GRM was calculated as $\theta$ **MM’** where M is a matrix of marker scores with dimensions *n* individuals by *m* markers, and $\theta$ is a proportionality constant (Endelman and Jannink 2012). The GRM was computed with the *A.mat* function in the *rrBLUP* R package (Endelman 2011). Within the model, residual error was formed of two parts with the nugget being NIID and then a correlated error term for rows and columns (AR1 x AR1, autoregressive first order correlation structure) (Isik et al. 2017 pg 93; 217). A separate AR1 x AR1 structure was fit for each cycle-site-year combination (14 total combinations), with ASREML requiring a complete row column matrix, with any incomplete observations filled in with dummy variables. This model fit one BLUP per genet regardless of if a trait had been measured one or multiple times and will be referred to as combined analysis as all years and cycles of data observations were combined in one model. For some traits, convergence failed using the AR1xAR1 model, and a reduced model with no row and column error structure was fit. Equation 1 was also fit individually for each cycle-year combination by dropping terms for cycle-year combination and repeated measurements across years.

### Genomic profiling

All genets were profiled using genotyping-by-sequencing (GBS) using a two enzyme protocol as in Poland et al. (2012). DNA extraction and pooled 192-plex GBS libraries were prepared at Kansas State University with all sequencing conducted at Hudson Alpha, Huntsville, AL using Illumina HiSeq machines. Single nucleotide polymorphisms (SNPs) were scored using the TASSEL GBSv2 pipeline (Glaubitz et al. 2014) and the *Thinopyrum intermedium* draft genome reference sequence (prerelease access provided by *Thinopyrum intermedium* Genome Sequencing Consortium). The IWG draft genome reference includes three sets of seven chromosomes numbered 1–7 based on homology to the seven chromosomes of barley (Kantarski et al. 2016). Chromosomes corresponding to three homoeologous groups (subgenomes) of IWG were designated 1J-7J, 1S-7S, and 1V-7V based on homologies to possible diploid ancestors in the pre released *Thinopyrum intermedium* draft genome reference sequence (Thinopyrum intermedium v2.1 DOE-JGI, http://phytozome.jgi.doe.gov/). A total of 123,423 putative SNPs were identified across the 6,824 genotyped genets. SNP filtering was completed based on four criteria similar to previous work in IWG (Crain et al. 2021a). First, each SNP was aligned to only one unique location on one of the 21 main chromosomes. Second, a minimum read depth of 4 tags was required to call a homozygous genotype, while heterozygotes could be called with a minimum two contrasting tags for each SNP. If the minimum read depth threshold was not met, the SNP site was set to missing. Third, the maximum data missing per SNP was 70%. Fourth, SNPs must have had a minor allele frequency (MAF) greater than 0.01. If an individual genet had more than 95% missing data the genet was removed from further analysis. After filtering, this dataset consisted of a total of 6,517 genets and 23,611 SNPs where the average genet had 11,478 markers and each SNP was called on average in 48% of the genets (Supporting Information Figure S1). Markers were imputed with Beagle version 4.1 using the default parameters (Browning and Browning 2016).

### Linkage disequilibrium and genetic parameters

Linkage disequilibrium (LD) was evaluated using TASSEL version 5.2.3 (Bradbury et al. 2007) for all pairwise comparisons within each chromosome for markers with a MAF > 0.05 and percent missing < 50%. The Hill and Weir formula (Hill and Weir 1988) was fit using the *nls* function in R (R Core Team 2020) to describe the extent of genome and chromosome LD using *r*^*2*^. The greater the distance at which half of the maximum value of the fitted value occurred, or *r*^*2*^=0.1 was considered the extent of LD (Flint-Garcia et al. 2003). The fixation index *F*_*ST*_ (Weir and Cockerham 1984) was used to evaluate population differentiation among cycles and was calculated using the *diveRsity* R package (Keenan et al. 2013). Unimputed marker data were used to calculate *F*_*ST*_ and allele frequency statistics. Values of *F*_*ST*_ > 0.15 were considered evidence of population differentiation whereas *F*_*ST*_ < 0.05 was considered as no evidence of population divergence (Hartl and Clark 1997 pg 118–19).

### Genome-wide association analysis and QTL identification

The *GWAS* function in *rrBLUP* (Endelman 2011) was used to assess marker-trait associations for each set of phenotypic trait data both jointly and by cycle-year combination. The GWAS model is a mixed-linear model (Yu et al. 2006) with the form:

2 $$y = X\beta + Zg + S\tau + e$$

where ***y*** is an *n* × *1* vector of phenotypic observations (BLUPs from Eq. 1), ${\varvec{\beta}}$ is a *p* × *1* vector of fixed effects where *p* is the number of fixed effects for population structure, **X** is an *n x p* design matrix for fixed effects, ${\varvec{g}}$ is an *n* × *1* vector of random polygenic effects, ***Z*** is an *n x n* matrix that is the **GRM**, ${\varvec{\tau}}$ is the fixed effect for a given marker being tested and ${\varvec{S}}$ is an *n* × *1* vector of marker scores for the respective locus, ${\varvec{e}}$ is an *n* × *1* vector of random residuals. Population structure was accounted for by using the first six principal components (*p* = 6), and model compression used ‘population parameters previously determined’ (P3D) (Zhang et al. 2010).

A total of 23,611 markers were tested for each trait, and markers with a significance threshold above a 0.05 false discovery rate (FDR) (Storey and Tibshirani 2003) were considered significant. The FDR was calculated using a modified function in the *rrBLUP* R package (Endelman 2011). Plots were created using the qqman R package (Turner 2017). For each significant marker, marker effects were determined using the *lmekin* function from the *coxme* R package (Therneau 2020) following the analysis of Sehgal et al. (2020). Percent variance explained (PVE) was calculated following methods by Broman and Sen (2009 pg. 246). As there was often more than one significant marker on the same chromosome, we used a minimum gap threshold of 100 Mb between significant markers to distinguish and count unique QTL. Each unique QTL was identified by the marker with the highest logarithm of the odds (LOD) value. Then any other significant marker located within 100 Mb was added to the QTL, followed by looking for markers within 100 Mb of the QTL region. This process was repeated, allowing for single QTL to progressively increase in size until there were no other significant markers located within 100 Mb of the endpoints of the QTL. The other significant markers not separated by at least 100 Mb are herein referred to as associated markers. If a chromosome had other significant markers that were outside of the original 100 Mb QTL region, a second QTL was declared.

Power analyses were completed using scripts from Wang and Xu (2019) where $\beta$ was set to 0.8. Minimum detectable QTL effect sizes were determined based on sample size, relationship between individuals, and heritability, where heritability was estimated from variance components of Eq. 1 as:

3 $$h^{2} = \frac{{\sigma_{g}^{2} }}{{\sigma_{p}^{2} }}$$

where $\sigma_{g}^{2}$ is genet variance, $\sigma_{p}^{2}$ is phenotypic variance which is the sum of genet variance, variance due to multiple observations, and residual error variance. The total number of QTL per trait was estimated using a squared exponential distribution from Hall et al. (2016) according to the formula:

4 $$n_{{{\text{QTL}}}} = \frac{{h^{2} }}{{\mu_{d} - \sqrt {2\mu_{d} \theta - \theta^{2} } }}$$

where $h^{2}$ is the heritability calculated from Eq. 2, ${\mu }_{d}$ is the average percent variance explained by detected QTL, and $\theta$ is the lowest detectable QTL estimated from the power analysis. In addition to using Eq. 4 for all traits with detected QTL, we estimated the minimum number of QTL for every trait by dividing 1 by the smallest detectable QTL size. The smallest detectable QTL size was obtained from the power analysis, and this analysis provided a lower bound on the number of QTL. Combining the power analysis, which provided a minimum detectable PVE, the number of estimated QTL (Eq. 4), and heritability we could estimate the size of the population required to detect QTLs explaining a given level of the total genetic variance (Lynch and Walsh 1998; Hall et al. 2016). For all analyses, we estimated the population size needed to detect QTLs accounting for 50% of the genetic variation.

### Data availability

All DNA sequence data has been deposited in the NCBI Sequence Read Archive (SRA) (https://www.ncbi.nlm.nih.gov/bioproject/) as part of the umbrella BioProject PRJNA609325. All scripts for data analysis and phenotypic data have been placed in the Zenodo digital repository: https://doi.org/10.5281/zenodo.6514719.

## Results

We assessed four TLI IWG breeding cycles that comprised approximately 4200 genets and five years of phenotypic data to dissect quantitative traits and inform breeding decisions. A linear mixed model was used to account for multiple years of phenotypic observations and calculate BLUPs, leveraging data collected within the breeding program to better understand IWG improvement. While a total of 34 different traits were observed across years (Supporting Information Table S1), the primary traits of selection were shattering, free-threshing seed, seed mass, and spike yield. Across cycles, seed mass and shattering were positively correlated with spike yield, while a negative association was generally observed between free threshing and spike yield (Supporting Information Figs. 2–5).

### Linkage disequilibrium analysis

**Fig. 1:**
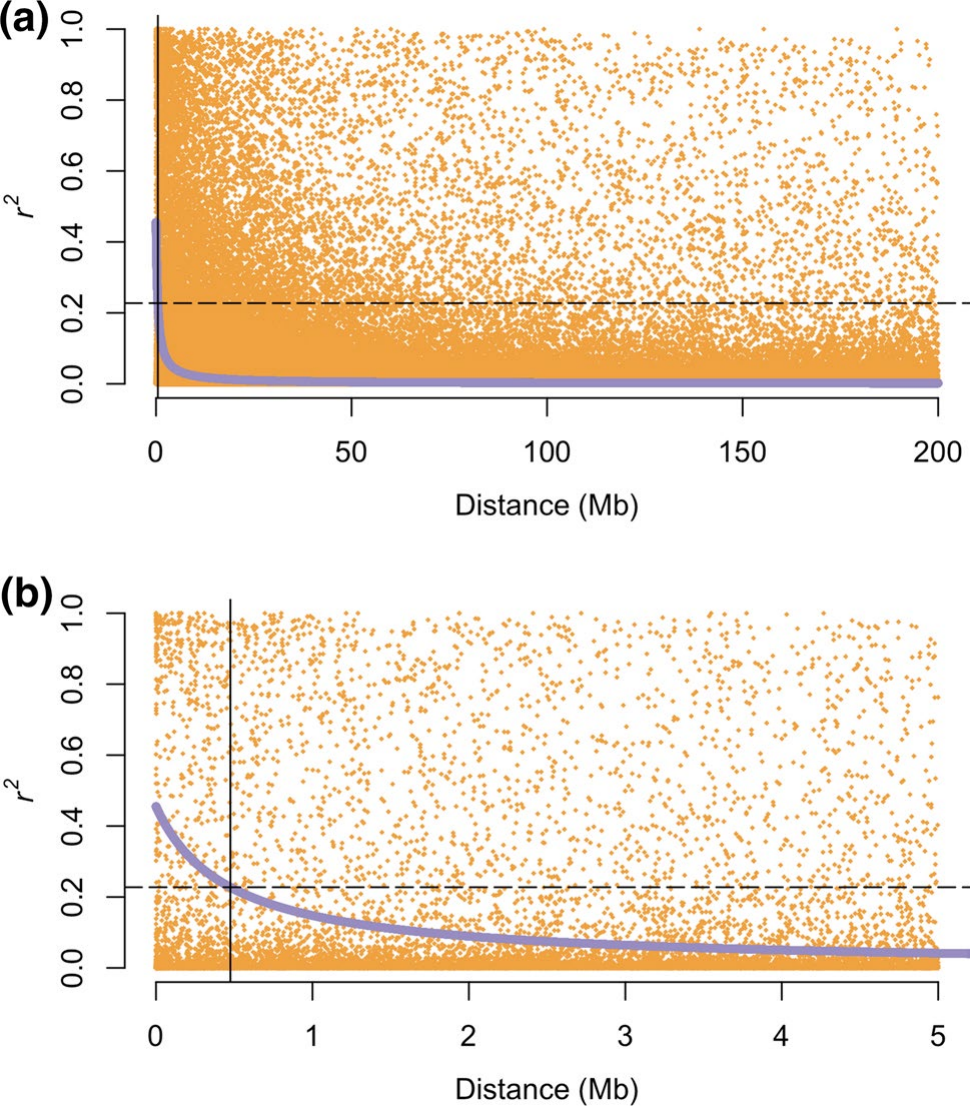
Genome-wide linkage disequilibrium (LD) for intermediate wheatgrass (*Thinopyrum intermedium*) for 200 Mb regions (**a**) and 5 Mb regions (**b**). Orange points represent individual marker combinations with a 250-marker sliding window. Average LD has been computed with the Hill and Weir formula (1988) and shown in blue. Vertical line represents the distance at which half-decay value occurs, with the dashed horizontal line showing the half-decay value

We evaluated the extent of linkage disequilibrium (LD) for markers across each chromosome. Within the breeding population, LD declined relatively rapidly, with genome-wide LD extending an average of 375 kb (Fig. 1). For individual chromosomes, the half decay distance for *r*^*2*^ ranged from less than 1 kb up to 1.43 Mb with chromosome 3S having the shortest LD and chromosome 3J the longest LD (Supporting Information Fig. 6). Even though average LD declined rapidly, there were numerous marker combinations that maintained LD at larger distances up to 50 Mb (Fig. 1). LD was not static across chromosomes and varied with location within chromosomes with centromeric regions showing much larger LD than telomeric regions (Supporting Information Fig. 7).

### Genome-wide association analysis

**Fig. 2:**
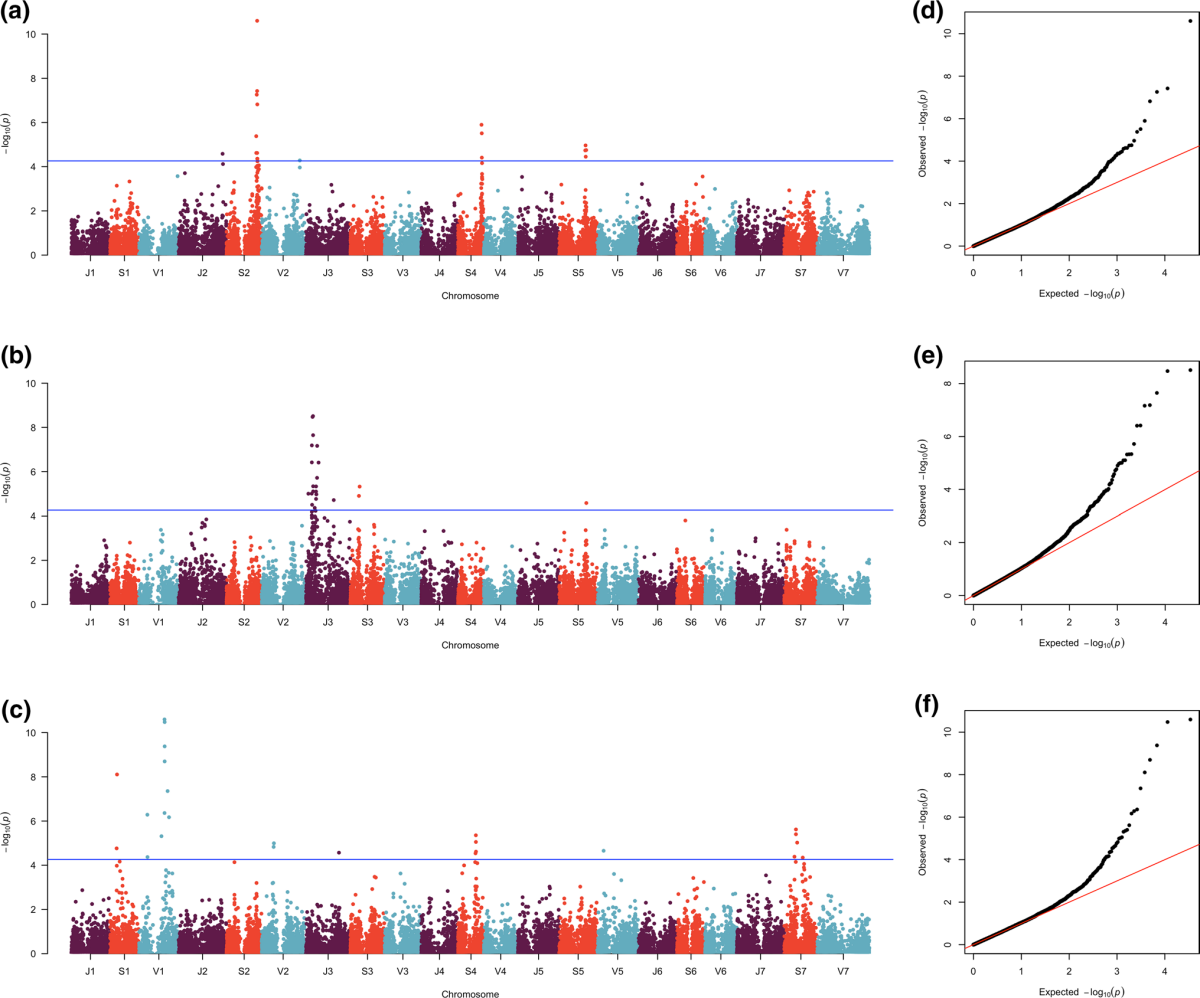
Manhattan plots of **a** shattering, **b** brittle rachis, and **c** seed circularity in intermediate wheatgrass (*Thinopyrum intermedium*) with line indicating 0.05 false discovery rate. Panels **d**–**f** show quantile–quantile (QQ) plots for p values under the null hypothesis (no association) and observed p values for shattering (**d**), brittle rachis (**e**), and seed circularity (**f**), respectively

We used a genome-wide association analysis to identify the location, number, and size of QTL underlying traits of interest to the IWG breeding program. Across all traits, the combined analysis found 93 marker-trait associations for 16 different traits, representing 37 separate QTL (Supporting Information Table 2). Of the traits of most interest to the breeding program—spike yield, free threshing, seed mass, and shattering—QTL were only identified for shattering (floret and brittle rachis) and free threshing. Both brittle rachis and free threshing had more than one QTL on the same chromosome (3J and 2V respectively, Table 1 and Fig. 2). The QTL effects were small explaining 1.0–2.7% of the observed phenotypic variation. The allele effects for identified QTL in shattering ranged from − 0.13 to 0.11 units less shattering on a 5-point scale. For free threshing, a reduction of up to 4.6 percentage points on a 100 point scale was observed for the alternate alleles compared to the reference genome (Table 1 and Supporting Information Table S2).

**Table 1:** Significant quantitative trait loci (QTL) associations in The Land Institute intermediate wheatgrass (*Thinopyrum intermedium*) breeding program for priority traits

Trait	QTL #	Associated markers	Chr	Position	*n*	LOD	F_ST_	PVE	Ref/alt	MAF	Ref value	Ref SE	Alt value	Alt SE	Het value	Het SE
Brittle rachis	1	17	3J	122,986,862	1441	8.51	0.01	2.7	A/G	0.38 (A)	0.93	0.008	0.04	0.009	0.02	0.007
	2	0	3J	421,522,694	1050	4.71	0.08	2.0	G/T	0.37 (T)	0.98	0.008	− 0.05	0.014	− 0.02	0.008
	1	1	3S	132,131,062	1653	5.32	0.06	1.5	C/G	0.34 (G)	0.95	0.005	0.05	0.009	0.03	0.005
	1	0	5S	391,487,396	1659	4.58	0.00	1.3	T/C	0.34 (T)	0.98	0.008	− 0.03	0.005	− 0.01	0.005
Free threshing	1	0	2V	196,007,664	2867	5.95	0.08	1.0	G/T	0.20 (T)	55.53	0.158	− 3.34	0.649	− 1.50	0.351
	2	0	2V	357,856,894	1478	6.11	0.12	1.9	T/G	0.18 (G)	55.93	1.103	− 4.65	0.963	− 1.79	0.846
Shattering	1	0	2J	636,725,449	1359	4.58	0.00	1.5	C/T	0.17 (T)	2.19	0.010	− 0.13	0.039	− 0.08	0.021
	1	6	2S	441,397,840	3130	10.60	0.01	1.5	T/C	0.47 (T)	2.13	0.015	0.10	0.008	0.04	0.009
	1	1	4S	341,952,545	2204	5.90	0.22	1.2	G/C	0.46 (C)	2.20	0.023	− 0.08	0.015	− 0.02	0.013
	1	2	5S	380,535,059	1620	4.96	0.00	1.4	T/A	0.25 (A)	2.16	0.030	0.11	0.027	0.08	0.023

Marker with highest LOD reported for each QTL; QTL #, number of QTL per chromosome; Associated markers, number of significant markers associated with each QTL; Chr, chromosome; *n*, number of individuals observed; *F*_*ST*_ fixation index between The Land Institute (TLI) Cycle 6 and TLI Cycle 9; PVE, percent variance explained; Ref/alt reference and alternate allele respectively; MAF, minor allele frequency for combined TLI Cycles 6–9 with minor allele in parenthesis; Ref value, reference allele value for a trait, Ref SE, standard error of reference value; Het, heterozygous

We found evidence for brittle rachis QTL which impacts shattering on chromosome 3J (Fig. 2), where the most significant marker (3J_122986862) was 5.6 Mb from IWG *brittle rachis 2* (*Btr2*) gene (Pourkheirandish et al. 2015) while another significant marker (3J_115931563, LOD = 8.47) was only 217 bp away from a *Btr2* gene. This QTL region was identified both in the combined analysis and analysis across individual cycles and years (Supporting Information Table 2) and was supported by up to 17 associated markers above the genome-wide threshold (Fig. 2) and constituted a 147 Mb region.

Seed circularity had the most significant markers of any trait in the combined analysis, with 23 associated markers representing eight unique QTL located across seven different chromosomes (Fig. 2, Supporting Information Table S2). For the number of florets per spike and florets per spikelet, one QTL region overlapped with colocalized markers on chromosomes 5J having the same directional effects (Supporting Information Table S2). One QTL for FSU was identified on chromosome 5S.

Along with analyzing the combined data, each cycle-year combination was analyzed independently. This resulted in 209 significant markers representing 67 unique cycle-year QTL being observed across 20 different traits (Supporting Information Table S2). Many of these QTL had several, up to 26, associated markers per QTL. Taken together, all analyses revealed QTL associations across 19 of the 21 chromosomes of IWG, with many chromosomes harboring QTL for multiple traits (Supporting Information Table S3).

Across 34 traits and up to nine cycle-year combinations, all the identified loci using the joint analysis explained minimal variation, with 5.2% percent variation explained (PVE, stem diameter) being the maximum for any combined analysis with an average of 1.7% PVE per identified QTL. When considering markers identified by cycle-year analysis, the PVE was greater than the combined analysis, yet only 14 of the 209 markers had PVE > 10%.

### Number of effective QTL

To evaluate the genetic architecture of these domestication and agronomic traits, we estimated the number of effective QTL for each trait using results from our power analysis, heritability, and QTL analysis. In general, our analysis of this breeding germplasm had the ability to detect small QTL, with PVE of the smallest detectable QTL ranging from 0.7 to 3.0% for each trait (Table 2). Determining the smallest detectable QTL also provided a lower bound estimate of the minimum number of QTL for each trait which ranged from 33 to 149 (Table 2) regardless of whether we had detected QTL. For traits with detected QTLs, we estimated the number of QTL for a given trait (using Eq. 4) which ranged from 93 to 357 (Table 2) for combined analysis. Using each cycle-year combination, a range of QTL could be estimated for traits with detected QTL. For important traits such as shattering, the estimated number of QTL ranged from 97 to 258, brittle rachis could be controlled by up to 293 QTL, and free threshing could have as few as 39 QTL. While the reported number of QTL could vary greatly within and between traits, these estimates demonstrate that these traits are highly polygenic and controlled by many loci.

**Table 2:** Number of estimated quantitative trait loci (QTL) for phenotypic traits in intermediate wheatgrass, and the estimated population size needed to detect QTLs explaining 50% of the genotypic variance

Trait	Smallest detectable QTL in percent variance explained	Minimum number of QTL	QTL estimate based on Hall et al. (2016)	Minimum QTL for individual cycle year combinations	Maximum number of QTL for individual cycle year combinations	Number of cycle-year combinations with observed QTL	h^2^	Minimum population size	Maximum population size
Brittle rachis	0.9	111	166	18	293	4	0.44	624	7339
Flag leaf height (cm)	3.0	34					0.44	1042	
Flag leaf length (cm)	2.8	36		5		1	0.31	212	1522
Flag leaf width (mm)	2.8	36		84		1	0.62	755	1633
Free threshing	0.7	147	304	39		1	0.63	809	5343
Lodging	1.3	76					0.38	2433	
maturity	0.7	147		6	221	2	0.50	281	5055
Number of florets per spike	0.9	110	214				0.23	5592	10,380
Number of florets per spikelet	0.9	110	177				0.25	5057	7919
Peduncle width (mm)	2.4	42	289	303		1	0.52	1004	6472
Floret site utilization	0.9	109	236	101		1	0.75	1572	3578
Plant height (cm)	0.7	149		14	558	3	0.38	627	15,931
Seed area (mm^2^)	0.7	147		4	22	2	0.69	93	2428
Seed density	0.7	147	133				0.45	3398	3737
Seed image circularity	0.7	144	239	16	88	4	0.52	475	4948
Seed length (mm)	0.7	147	251	113		1	0.58	2298	4864
Seed mass (mg)	0.7	147					0.63	2732	
Seed perimeter (mm)	2.7	37					0.79	613	
Seed width (mm)	0.7	147	126	14	115	3	0.36	628	4550
Seeds per spike	0.7	148					0.42	3972	
Shattering	0.7	148	187	97	258	6	0.40	2823	7139
Spike dry weight (g)	0.9	110					0.36	3625	
Spike emergence (cm)	2.3	43	283	266		1	0.51	1064	6102
Spike emergence %	1.4	74	357				0.51	1770	7645
Spike harvest index	0.9	110					0.32	3961	
Spike length (cm)	1.3	78	268	547		1	0.49	1890	12,297
Spike yield (g)	0.7	148					0.53	3231	
Spikelet density	2.7	37		21		1	0.63	482	777
Spikelets per spike	0.9	110	101	14		1	0.25	948	5153
Stem angle	1.3	79					0.59	1586	
Stem diameter (mm)	2.9	35	93	50		1	0.51	897	2197
Stem strength bottom	3.0	33		30		1	0.45	934	979
Stem strength middle	1.3	75		8		1	0.30	515	3049
Stem strength top	3.0	33					0.77	552	

Maximum population size only calculated if more than one number of QTL estimate available

We also estimated the population size that would be required to detect QTLs explaining 50% of the genetic variation. Population size differed between traits, ranging from a minimum of 98 up to 15,931 plants with an average population size of 1720 (Table 2). For priority breeding traits of spike yield, reduced shattering, and seed mass, the minimum population sizes, to detect QTLs explaining 50% of the genetic variance, were all > 2500 plants.

### Allele frequency and *F*_*ST*_

Using all markers, fixation index, *F*_*ST*_*,* was calculated to examine the potential impact of directional selection which would generate genetic differentiation between TLI-Cycle 6 and 9. Only 548 (2.3%) of these markers showed high or very high genetic differentiation, suggesting that many areas of the genome have not been under consistent selection pressure (Table 3). Of the 72 markers that showed very high genetic differentiation, they were distributed across 20 of the 21 chromosomes. For significant loci identified by GWAS, allele frequency and *F*_*ST*_ were used to further evaluate changes between TLI breeding Cycles 6 and 9. As direct selection should alter allele frequency, we expected this analysis to provide evidence of selection pressure. Of the significant QTLs identified, only three markers (4.5%) had *F*_ST_ values > 0.15 (Table 4, Supporting Information Table S2).

**Table 3:** Fixation index, *F*_*ST*_, values for all single nucleotide polymorphisms (SNPs) in The Land Institute intermediate wheatgrass breeding population cycles 6 and 9

Level of differentiation	Range of *F*_*ST*_ values	Number of loci (%)			
		Genome-wide	Shattering	Seed circularity	Brittle rachis
Little	0.00—0.05	18,258 (77.3)	26 (83.9)	26 (60.4)	44 (78.6)
Moderate	0.05—0.15	4805 (20.3)	3 (9.7)	16 (37.2)	12 (21.4)
High	0.15—0.25	476 (2.0)	2 (6.4)	1 (2.3)	0
Very High	> 0.25	72 (0.3)	0	0	0
Total		23,611	31	43	56

Level of differentiation of *F*_ST_ values is from Hartl and Clark (1997)

**Table 4:** Biallelic reference allele frequency for four breeding cycles in The Land Institute breeding program that had Fixation index, *F*_*ST*_*,* greater than 0.15

Trait	Marker	Cycle 6	Cycle 7	Cycle 8	Cycle 9
Shattering	4S_341952545	0.74	0.58	0.42	0.40
Shattering	4S_349886731	0.74	0.51	0.41	0.45
Seed circularity and seed length	1V_438389996	0.69	0.78	0.84	0.96

**Fig. 3:**
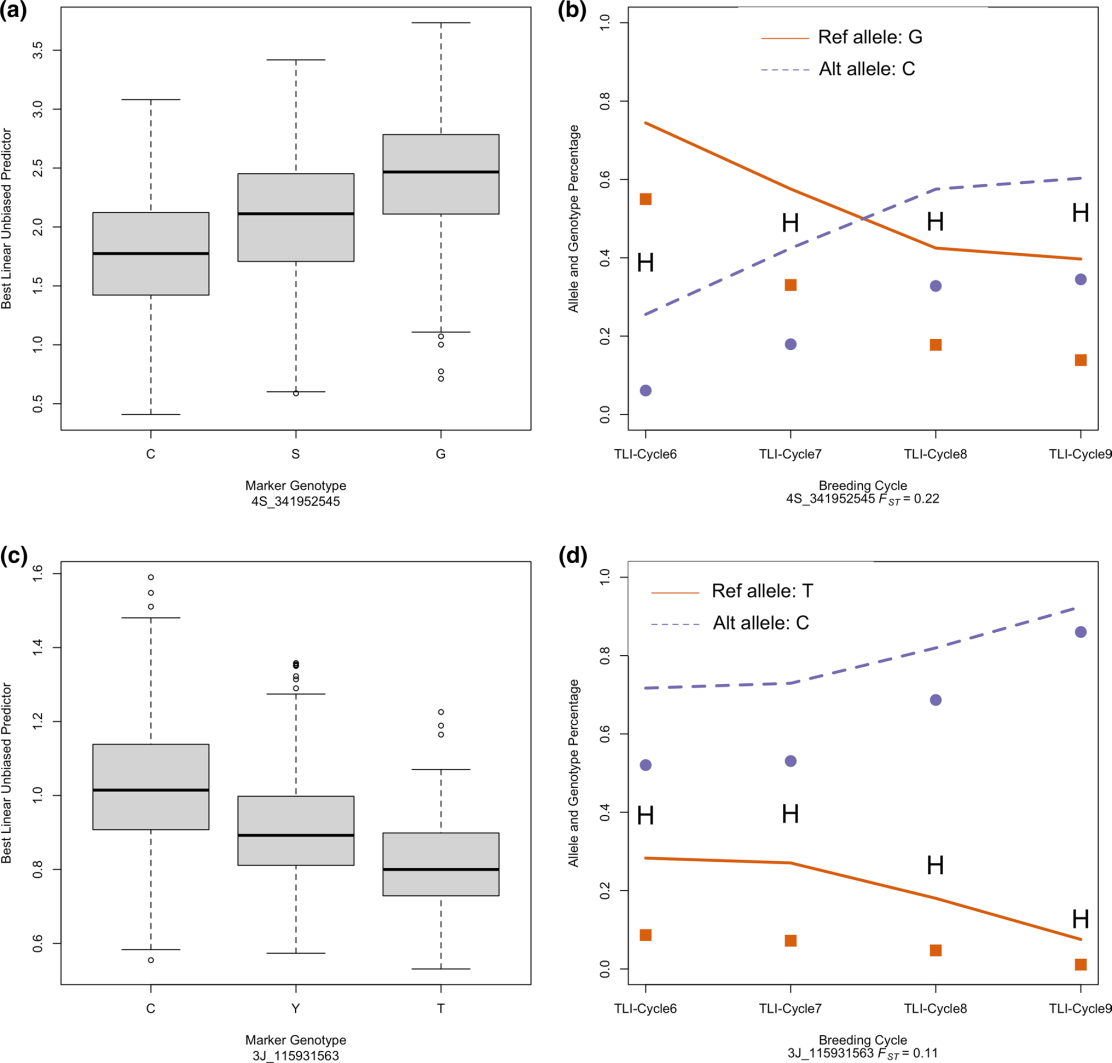
Distribution of phenotypic values for shattering and brittle rachis (**a** and **c** respectively), where lower values are preferred, at the marker loci 4S_341952545 and 3J_115931563 in intermediate wheatgrass (*Thinopyrum intermedium*). Panels **b** and **d** display the allele (line plots) and genotype (points, H is heterozygote) frequency change for the shattering marker in The Land Institute (TLI) Cycle 6 to 9 breeding populations; population differentiation expressed with *F*_*ST*_ between TLI Cycle 6 and TLI Cycle 9

Of 24 traits with QTL, only three traits—seed circularity, shattering, and seed length—showed *F*_*ST*_ > 0.15 for any single trait-associated marker. For shattering, a trait that has been under strong selection, two markers on chromosome 4S had high *F*_*ST*_ values (Fig. 3) and the other associated markers on chromosome 4S had moderate *F*_*ST*_ values. For this trait, all other significant markers on chromosomes 2J, 1S, 2S, 3S, 5S, and 2V did not show any significant differentiation (*F*_*ST*_ < 0.05) after 3 cycles of selection based on *F*_*ST*_ values. The high *F*_*ST*_ value of marker 4S_341952545 resulted in the alternate allele frequency increasing from 26 to 60%, corresponding to a -0.08 unit decrease in shattering over three cycles of selection (Fig. 3, Table 4). Compared to the 2.3% of genome-wide markers that showed high differentiation, up to 6.4% of the shattering markers had high *F*_*ST*_ values.

The marker 3J_115931563, which had a strong association with brittle rachis, had a moderate *F*_*ST*_ value of 0.11. Surprisingly, from TLI-Cycle 6 to TLI-Cycle 9 the frequency of the favorable (reference) allele decreased from 28 to 7.5%, in the opposite direction as expected by selection for reduced shattering. Evaluating this locus for all other traits showed that the reference allele while favorable for shattering and free threshing was detrimental to spike yield, seeds spike^−1^, and spike dry weight. This suggests that there is tradeoff between spike yield and brittle rachis at this locus and that selection for increased yield could be driving the alternate allele.

In addition to shattering, high *F*_*ST*_ values were observed for seed circularity and seed length at the locus 1V_438389996. This marker showed the reference allele frequency changed from 0.69 to 0.96% over the four cycles, corresponding to a decrease in seed circularity and increase in seed length, i.e. a more elongated seed. Even though significant markers were identified for number of florets per spike, florets per spikelet, spikelets per spike, and FSU—yield component traits—all markers except for one showed little differentiation across the four cycles of selection. Even for the priority trait of free threshing, only two of 10 markers (20%) showed moderate differentiation. The *F*_*ST*_ values for markers associated with plant height, which has not been targeted by selection, did not show any differentiation.

## Discussion

### GWAS in a breeding population

The TLI IWG breeding program has completed four cycles of breeding since 2017 using GS with current evidence suggesting that genetic gains can exceed 8% year^−1^ for spike yield and up to 14% year^−1^ for free threshing (Crain et al. 2021a). Given the magnitude of the challenges to domesticate a new crop, identifying genomic regions controlling traits should be a priority within breeding programs to accelerate gains. As such, we used breeding data to complete GWAS analysis for 34 traits. While many markers were identified as QTL, there appeared to be little consistency from one cycle-year combination to the next, suggesting genotype-by-environment interaction between years. Additionally, the PVE explained by markers evaluating cycle by year combinations was generally higher than the combined analysis, ranging from 2 to 15%, suggesting these model estimates are upwardly biased because of truncation of QTL below the detectable threshold (Beavis 1994; Xu 2003). When all data were analyzed together, the addition of genotype-by-environment interaction limited our ability to identify genomic signals.

Brittle rachis had significant MTAs, but until 2019 this trait was not considered separate from shattering and not measured extensively in the population until 2020. As this marker, 3J_115931563 is associated with a known gene—*Btr2*—and has been found in other IWG populations (Altendorf 2021b), it is a prime candidate for the TLI breeding program to select and drive to fixation over the next few cycles. While our data did not show this locus associated with any other trait, work by Larson et al. (2019) identified traits including spikelets spike^−1^, seeds spikelet^−1^, seed area, and spike length on this chromosome which could explain the reduction of the favorable allele for brittle rachis.

Even though we evaluated 34 traits, very few significant associations were found, including for spike yield and seed mass, which have been a primary target of selection. Leveraging a large number of observations, we estimated the effective number of QTL in the population. While the estimations hinge on a number of assumptions including QTL distribution (Otto and Jones 2000), additive genetic effect, and random segregation (Hall et al. 2016), they provide an approximation of the complexity of given traits. Considering the ability of our analysis to detect small QTL, as small as only 0.7% of the phenotypic variation, and a large number of minimum QTL estimated, there appear to be no large effect QTL that can be targeted by marker-assisted selection for IWG improvement. The apparent deficiency of large effect QTL could indicate that the intense selection bottleneck of IWG from 14 plants (Zhang et al. 2016) essentially fixed major allele effects in early generations or that large effect genes were not in the founder population. Even though research shows estimated genetic gains for spike yield of up to 8% year^−1^ in TLI-Cycle 7–8 (Crain et al. 2021a) it appears that these gains are from small effect loci. It should be noted that selection within the breeding program was based on GS values and not on any particular marker per se. This information suggests that the current breeding material has highly polygenic traits that follow an infinitesimal model (Fisher 1918; Barton et al. 2017). Interestingly, we determined that 2.3% (548 markers) had diverged between TLI-cycle 6 and 9, yet only four of the 217 MTAs showed high or very high *F*_ST_. Since genomic selection has been exclusively used for the last four breeding cycles (Crain et al. 2021a), we evaluated the genome-wide estimated marker effects for yield traits. Of the 72 highly diverged markers, 60% and 56% (43 and 40 markers respectively) had coefficients with higher spike yield and seed mass and the mean of all these markers indicated selection was in the favorable direction. As the IWG breeding continues, improvement will be from selecting many small effect alleles.

### Linkage disequilibrium and allele dynamics

Linkage disequilibrium was evaluated across the IWG genome and is key to interpreting the GWAS results and the estimated number of QTL. Within this IWG population, LD declines rapidly relative to closed breeding populations and half-decay rarely exceeded 1 Mb for any chromosome. In a separate IWG breeding population, Bajgain et al. (2021) estimated LD was 406 kb, thus in general agreement to this study. In comparison to bread wheat breeding populations which have LD estimated to be 50 Mb (Juliana et al. 2018), LD is lower, necessitating more markers to cover the genome. If markers were evenly spaced over the 12.75 Gb IWG genome (Vogel et al. 1999) a minimum of 27,000 markers would be needed to ensure all parts of the genome were in LD with a marker to increase mapping resolution. While we leveraged a high-quality draft genome reference sequence, our results could be influenced by the current genome assembly. It is possible that some of the significant marker positions will change, providing a more complete picture of trait observations and LD dynamics.

The extent of LD is also highly variable across the genome. This is particularly true of the identified brittle rachis QTL on chromosome 3J where the LD block for the QTL region spanned greater than 100 Mb. It is well known that recombination is highly restricted in the centromeric regions of Triticeae chromosomes, thus it is expected larger linkage blocks, greater than 100 Mb can be found in the centromeres compared to the telomeric regions.

While LD is estimated to decline rapidly, it should be noted that many of our significant GWAS hits had multiple markers extending beyond the expected LD. This could indicate that selection has created larger linkage blocks. In maize, LD in diverse lines is estimated to be less than 1 kb (Tenaillon et al. 2001) to over 100 kb in elite maize lines (Rafalski 2002), showing the extent that selection and narrow sets of germplasm can increase the LD block size. As GS has been the method of selection in cycles 7 through 9, this could be creating larger LD blocks for genomic regions with larger effects, thus under higher selection intensity. This would include both traits under direct selection such as spike yield as well as any trait that indirectly contributes to priority traits. It is also likely that there are different historical sources of LD, particularly from small effective population sizes, including LD created between the time of collection and the initial bottleneck of selection at the Rodale Institute, and LD created in the TLI breeding program (Zhang et al. 2016; DeHaan et al. 2018). While selection could be altering LD, these results could be influenced by the amount of missing data, which is common in GBS markers (Davey et al. 2011; Poland and Rife 2012) as well as the lack of phasing. As informatic methods improve, phasing or positioning SNPs relative to each chromosome could vastly improve our analysis of LD and GWAS resolution (Browning and Browning 2011, 2012).

### Comparison to other IWG GWAS studies

Within IWG, several other studies have evaluated important domestication and agronomic traits, providing corroboration of key results. Studies have shown that IWG has strong collinearity with the barley genome, (Kantarski et al. 2016; Zhang et al. 2016) providing resources to identify candidate genes. Within a nested association mapping panel, Altendorf (2021b) found the same marker as this study for brittle rachis. While this marker is closest to a *Btr2* gene, it is in a 7 Mb region with many *btr*-like genes (Pourkheirandish et al. 2015; Civáň and Brown 2017; DeHaan et al. 2020). Using a bi-parental IWG population, Larson et al. (2019) investigated QTL for domestication traits and found several overlaps with the current study. For seed shattering, Larson et al. (2019) discovered QTL on chromosome 2J and 4S that align with results found in this study. Chromosome 4S had the most significant seed shattering QTL (LOD > 15.0) in a full-sib family derived from C3_3471, which has been described as the first non-shattering and free-threshing IWG plant (Larson et al. 2019). There was also close alignment with free threshing QTLs located on chromosome 2V.

One of the most unanticipated results from this study was the large number of markers associated with seed circularity. One potential explanation is the effect of self-incompatibility genes, as these have been shown to have an impact on seed size and fertility in a full-sib mapping population of perennial ryegrass (*Lolium perenne* L.)(Studer et al. 2008). Self-incompatibility (SI) in grasses is controlled by a two-locus (*S* and *Z*) system (Lundqvist 1954; Cornish et al. 1979; Baumann et al. 2000), which are located on homoeologous groups 1 and 2 of IWG, respectively (Larson et al. 2019; Crain et al. 2020b). Self-incompatibility has been documented in IWG (Dewey 1978; Jensen et al. 1990), and previously reported markers for seed area, seed width, seed length, and seed weight by Zhang et al. (2017), Bajgain et al. (2019), and Larson et al. (2019) are located near putative *S* orthogenes on homoeologous group 1 of IWG. Although the mechanism of SI is not completely characterized, Manzanares et al. (2016) demonstrated that a domain of unknown function (*S-DUF247*) is involved in SI reactions. This region has also been associated with seed weight (Zhang et al., 2017; Larson et al. 2019), seed length (Bajgain et al. 2019), and was identified by Crain et al. (2020b) as an active SI locus in IWG. Regardless of whether the loci related to seed circularity are related to potential SI activity or putative control of seed circularity, these loci could be beneficial to the breeding program because seed shape could have an impact on milling quality. Marshall et al. (1984) proposed that spherical seeds maximize volume to surface ratio. IWG has very long and thin seeds (Zhang et al. 2017), so selecting loci that alter seed shape could be used to both improve yield and end-product use.

### Application to improving IWG

While we did not find any large effect QTL, our results suggest several potential applications within the breeding program. Our data support that continued use of GS models for breeding and selection is appropriate. While Bajgain et al. (2019) suggested using QTL as fixed effects in GS models to improve predictions, none of our detected QTL explained more than 10% of the variance, which would be large enough to be included as a fixed effect as suggested by Bernardo (2014). Second, based on the QTL effect size, genetic mapping studies will require large population sizes to accurately identify and estimate QTL. While the breeding program routinely analyzes 4,000 plants, this is probably the smallest number of plants needed for genetic mapping based on our power analysis and assessment of genetic architecture for these key traits. Lastly, selection pressure on traits that would indirectly contribute to yield, could be adjusted to ensure efficient plants, such that spike yield is not increased at the expense of larger, less efficient spikes with lower FSU. Even though MTAs were identified for some of these traits, current results showed minimal allele differentiation between TLI-Cycles 6 and 9. The observed phenotypic variation of these traits suggests that GS can continue to be an effective tool to improve these traits. Along with FSU, biomass production (Vico et al. 2016) and seed set (Armstead et al. 2008) have been suggested as important steps in increasing perennial seed production.

By utilizing data generated within the breeding program, this study identified MTA for several agronomic and domestication traits, all of which had small effects suggesting that traits are highly polygenic. Even for traits like brittle rachis that are expected to be controlled by a few major genes, we estimated more than 100 QTL control this trait. In comparison to systems like barley, where brittle rachis is controlled by a well-defined two-locus system (Pourkheirandish et al. 2015), our results indicate that many traits are under polygenic control. While GWAS is traditionally used to identify quantitative traits, work by Bandillo et al (2017) showed that GWAS could identify qualitative genomic regions in soybean. This result suggests that improvement of these traits and domestication, even though well defined in annual crops, maybe more challenging than simply fixing a small number of loci and needs to be thought of as an iterative process (Van Tassel et al. 2020). Within IWG and new crop development, this finding could also provide insight into future biotechnology solutions such as genome editing (DeHaan et al. 2020). Alternatively, simple control of these traits by major genes may be possible, but the necessary genetic variation may be lacking from the breeding program, or obtaining the correct combination of recessive alleles may be difficult to achieve in an outcrossing polyploid species.

Even though no QTL were identified for spike yield and seed mass, several QTL were found for component traits of yield, suggesting that genetic control of these traits is from many small effect loci. Previous breeding efforts have increased spike yield by 77% and seed mass by 23% over two breeding cycles (DeHaan et al. 2014). These results, coupled with genetic gain estimates of over 8% year^−1^ and heritability estimates ranging from 0.32 to 0.66 by Crain et al. (2021a), suggest that the current breeding program has considerable genetic variance, providing opportunity for continued improvement well into the future. There appear to be few large effects QTL, with the substantial genetic progress having been made through selection of many loci across the genome with small effects. These observations support a continued focus on population improvement methods based on an underlying infinitesimal model of genetic architecture (Fisher 1918; Barton et al. 2017) and further implementation of genomic selection. The challenge of developing perennial grains is daunting, yet the knowledge generated from this study will help select high-yielding and high performing genets, leading to widely grown perennial grain crops.

## Supplementary Information

Below is the link to the electronic supplementary material.

Supplementary file1 Distribution of the number of markers (m = 23,611) per genet (n = 6,517) in panel (a), and the distribution of fraction of missing markers in each genet, panel (b) (PNG 136 KB)

Supplementary file2 Figure S2. Correlations of predicted breeding values for priority traits in The Land Institute breeding program Cycle 6 (PNG 585 KB)

Supplementary file3 Figure S3. Correlations of predicted breeding values for priority traits in The Land Institute breeding program Cycle 7 (PNG 591 KB)

Supplementary file4 Figure S4. Correlations of predicted breeding values for priority traits in The Land Institute breeding program Cycle 8 (PNG 579 KB)

Supplementary file5 Figure S5. Correlations of predicted breeding values for priority traits in The Land Institute breeding program Cycle 9 (PNG 618 KB)

Supplementary file6 Figure S6. Chromosome-wide linkage disequilibrium (LD) for intermediate wheatgrass (Thinopyrum intermedium) for 10 Mb region for each chromosome (panels a-u). Average LD has been computed with the Hill and Weir formula (1988) and shown in blue. Vertical line represents the distance at which half-decay value occurs, with the dashed horizontal line showing the half-decay value (PNG 3515 KB)

Supplementary file7 Figure S7. Heat map of linkage disequilibrium (LD) across chromosome 3J with 461 single nucleotide polymorphic markers. Upper triangle is R2 values colored according to the key on the right, with the lower triangle showing p-values colored according to scale below the x-axis (PNG 2184 KB)

Supplementary file8 Table S1 Descriptive statistics, including total number and number of genets by cycle, of the best linear unbiased predictors of 34 traits measured in The Land Institute intermediate wheatgrass breeding program cycles 6-9 during 2016-2020 (DOCX 19 KB)

Supplementary file9 Table S2 Significant marker-trait associations for The Land Institute intermediate wheatgrass breeding program analyzed by combined data and cycle-year combinations (XLSX 37 KB)

Supplementary file10 Table S3 Chromosome location of genome-wide associations by trait for combined analysis (C) and individual cycle combinations (6-9) for The Land Institute intermediate wheatgrass breeding program (DOCX 20 KB)

## Abbreviations

BLUP: Best linear unbiased predictor
FDR: False discovery rate
*F*_*ST*_: Fixation index
FSU: Floret site utilization
GBS: Genotyping-by-sequencing
GRM: Genomic relationship matrix
GS: Genomic selection
GWAS: Genome-wide association study
IWG: Intermediate wheatgrass
LD: Linkage disequilibrium
LOD: Logarithm of the odds
MTA: Marker-trait association
PVE: Percent variance explained
QTL: Quantitative trait loci
SNP: Single nucleotide polymorphism
TLI: The Land Institute

## Declarations

### Data availability

Original sequence data has been uploaded to the NCBI sequence read archive (SRA) (https://www.ncbi.nlm.nih.gov/bioproject/) as part of the umbrella BioProject PRJNA609325.

### Code availability

R code generated for this study has been deposited in the Zenodo digital repository https://doi.org/10.5281/zenodo.6514719.
