# Supplementary figures and images for: Genetic architecture and QTL selection response for Kernza perennial grain domestication traits
Source: Theor Appl Genet. 2022 Jun 28;135(8):2769–84. doi: 10.1007/s00122-022-04148-2 (PMC9243872; doi:10.1007/s00122-022-04148-2)

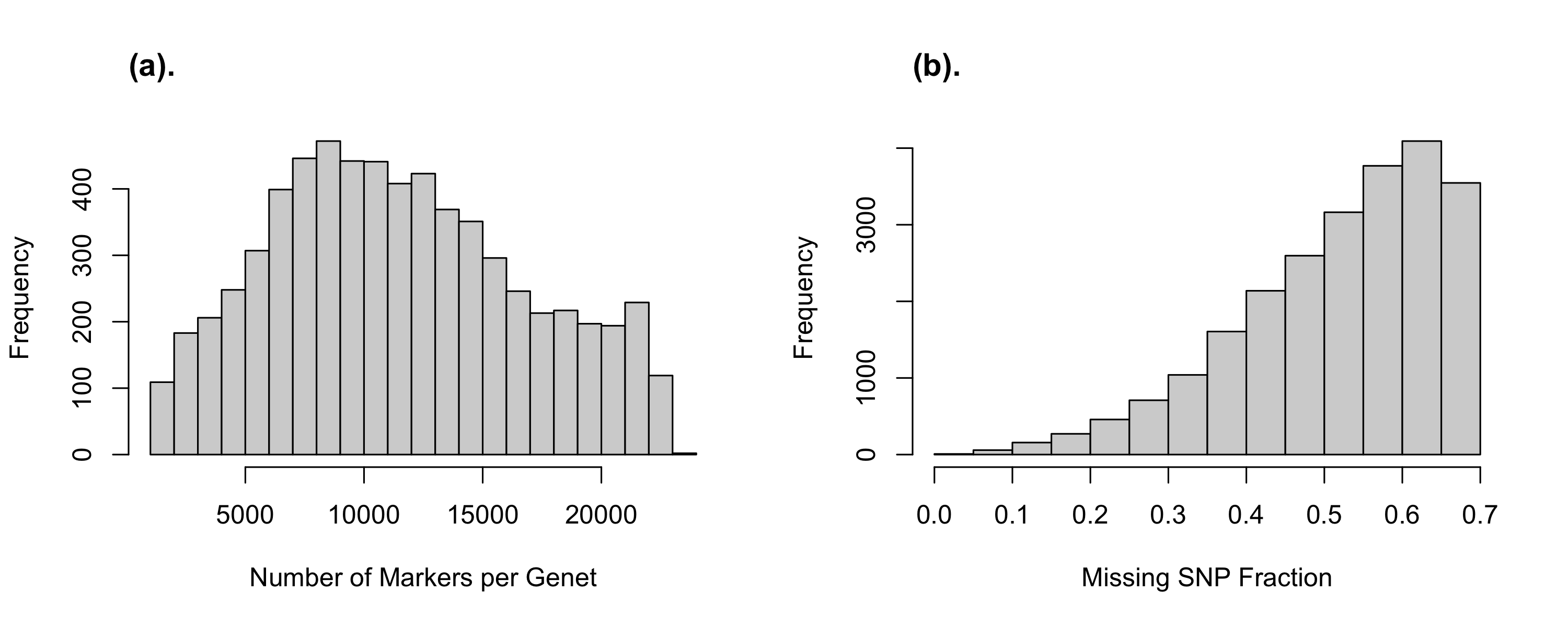

Supplement: Supplementary file 1 — Supplementary file1 Distribution of the number of markers (m = 23,611) per genet (n = 6,517) in panel (a), and the distribution of fraction of missing markers in each genet, panel (b) (PNG 136 KB) [file 122_2022_4148_MOESM1_ESM.png]

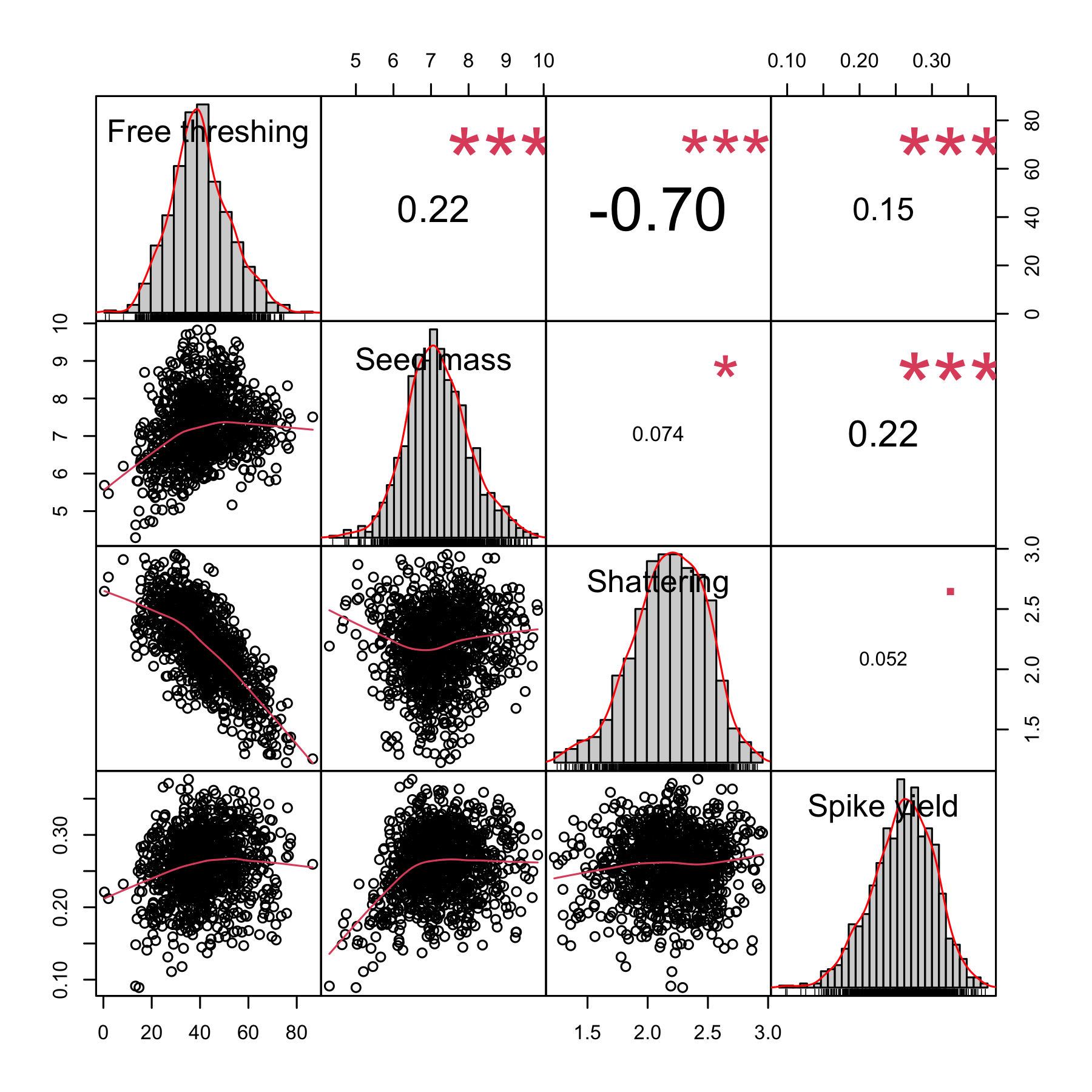

Supplement: Supplementary file 2 — Supplementary file2 Figure S2. Correlations of predicted breeding values for priority traits in The Land Institute breeding program Cycle 6 (PNG 585 KB) [file 122_2022_4148_MOESM2_ESM.png]

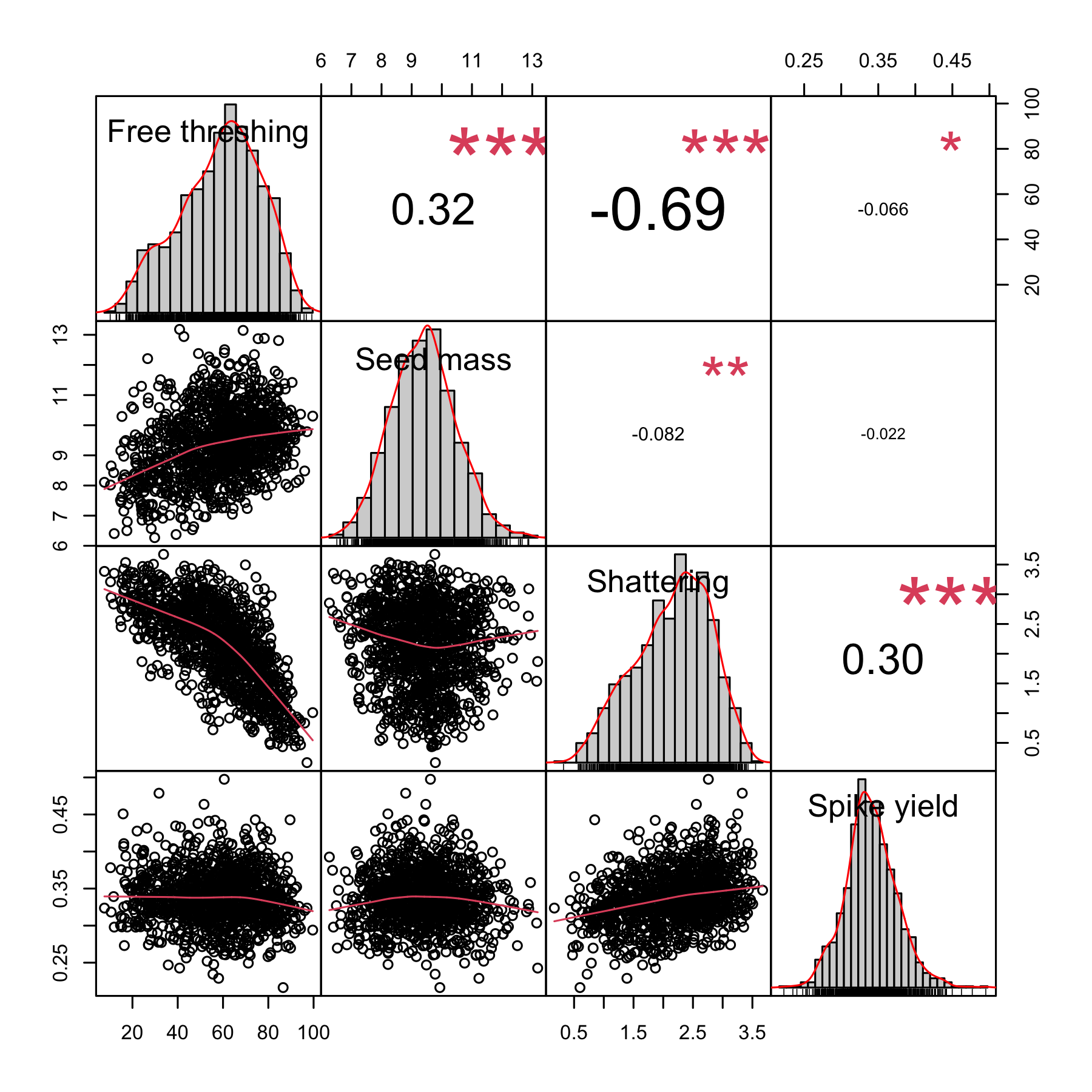

Supplement: Supplementary file 3 — Supplementary file3 Figure S3. Correlations of predicted breeding values for priority traits in The Land Institute breeding program Cycle 7 (PNG 591 KB) [file 122_2022_4148_MOESM3_ESM.png]

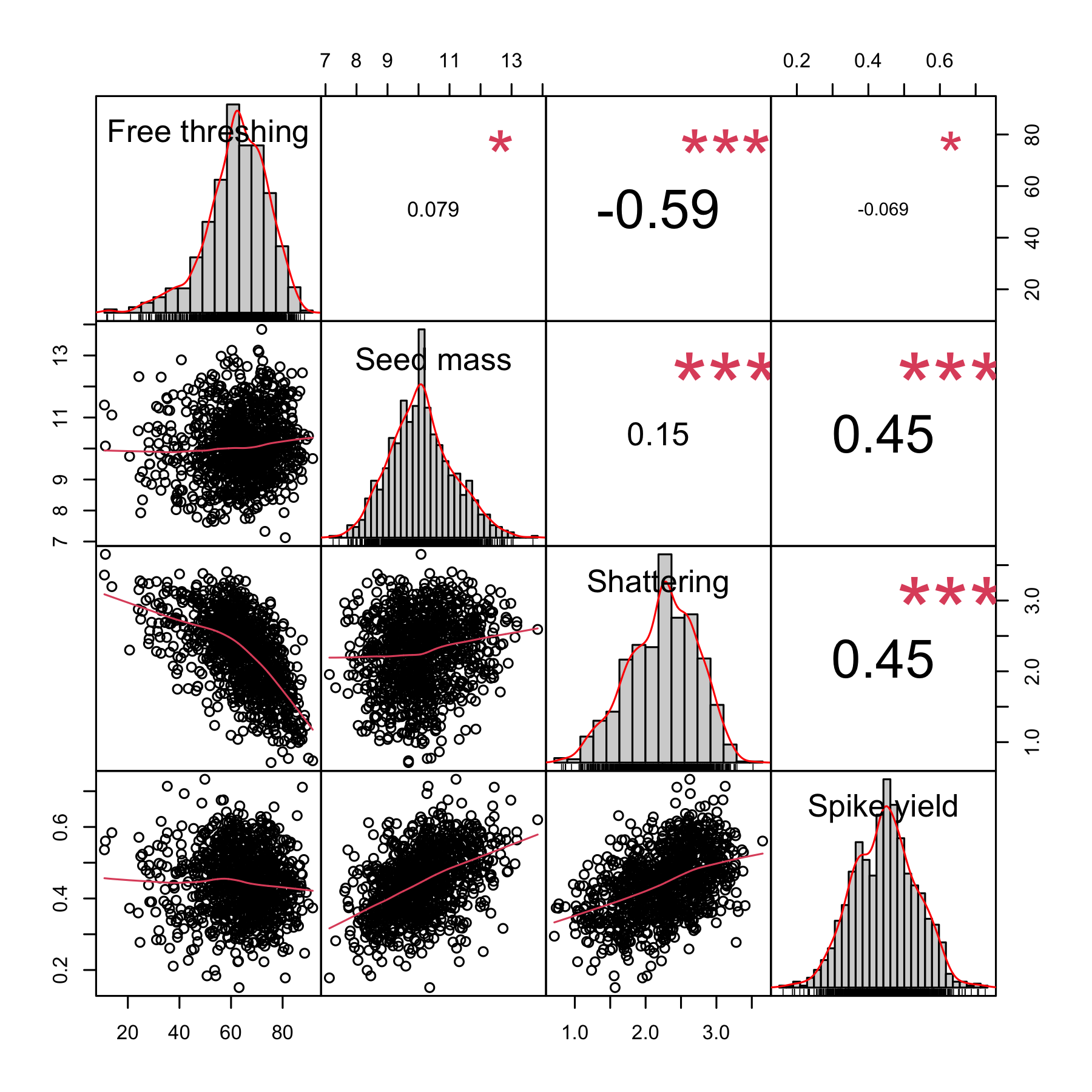

Supplement: Supplementary file 4 — Supplementary file4 Figure S4. Correlations of predicted breeding values for priority traits in The Land Institute breeding program Cycle 8 (PNG 579 KB) [file 122_2022_4148_MOESM4_ESM.png]

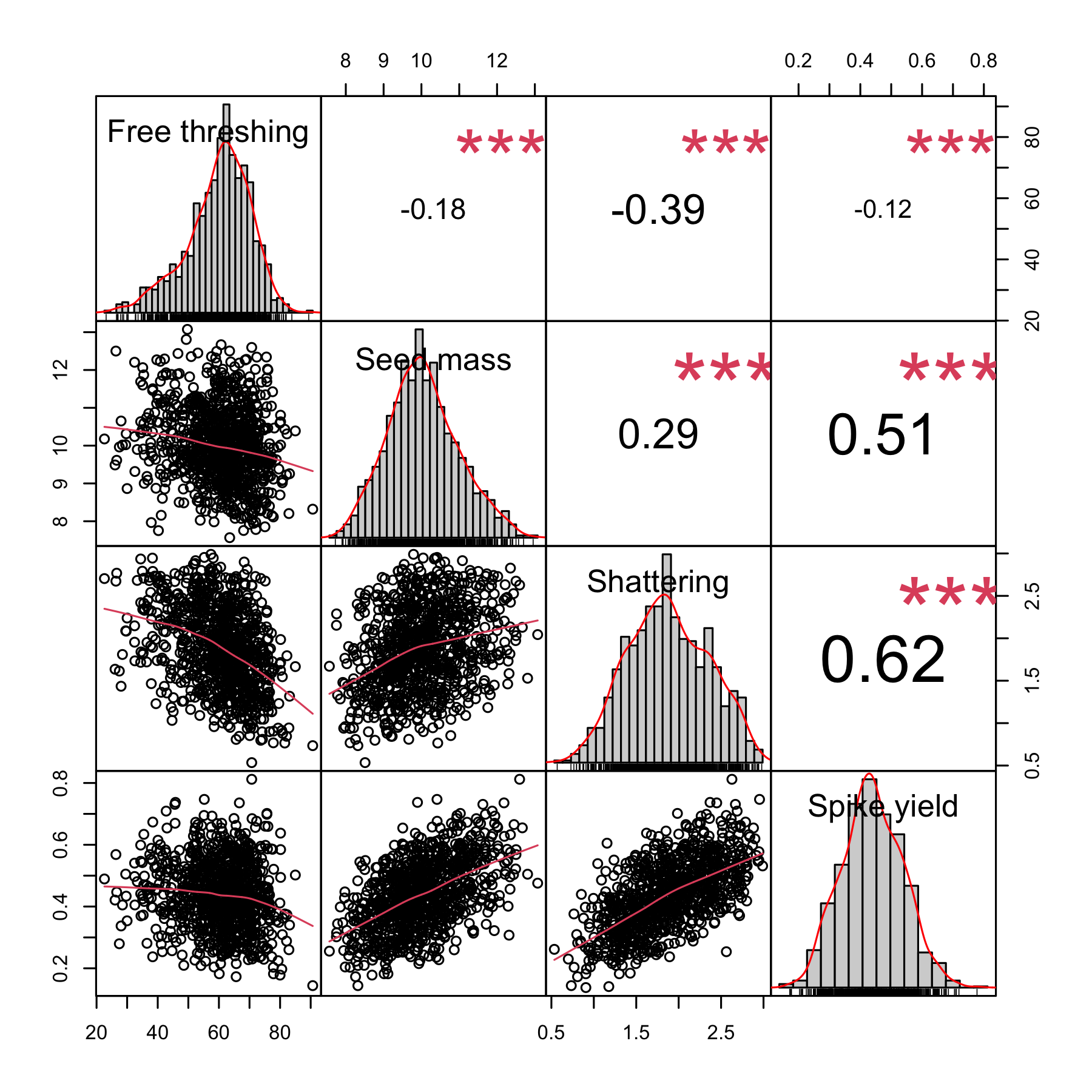

Supplement: Supplementary file 5 — Supplementary file5 Figure S5. Correlations of predicted breeding values for priority traits in The Land Institute breeding program Cycle 9 (PNG 618 KB) [file 122_2022_4148_MOESM5_ESM.png]

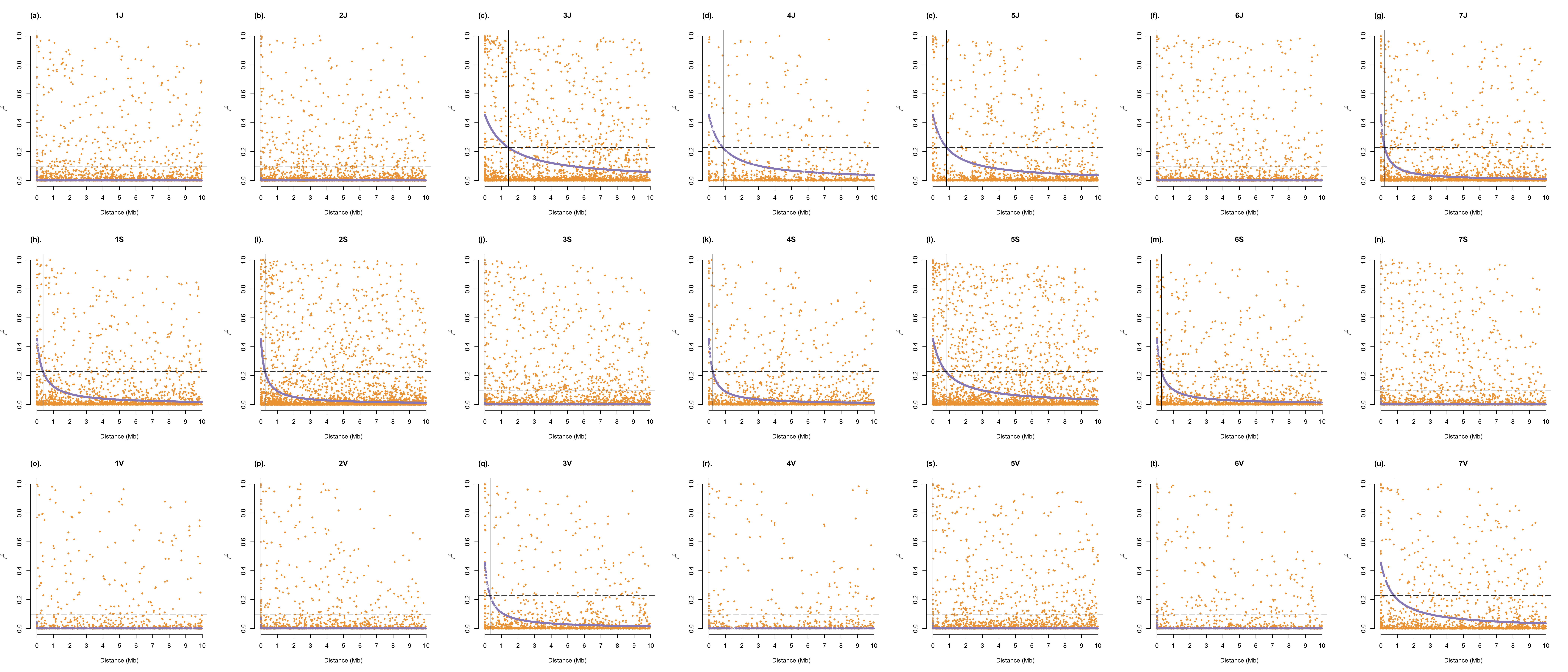

Supplement: Supplementary file 6 — Supplementary file6 Figure S6. Chromosome-wide linkage disequilibrium (LD) for intermediate wheatgrass (Thinopyrum intermedium) for 10 Mb region for each chromosome (panels a-u). Average LD has been computed with the Hill and Weir formula (1988) and shown in blue. Vertical line represents the distance at which half-decay value occurs, with the dashed horizontal line showing the half-decay value (PNG 3515 KB) [file 122_2022_4148_MOESM6_ESM.png]

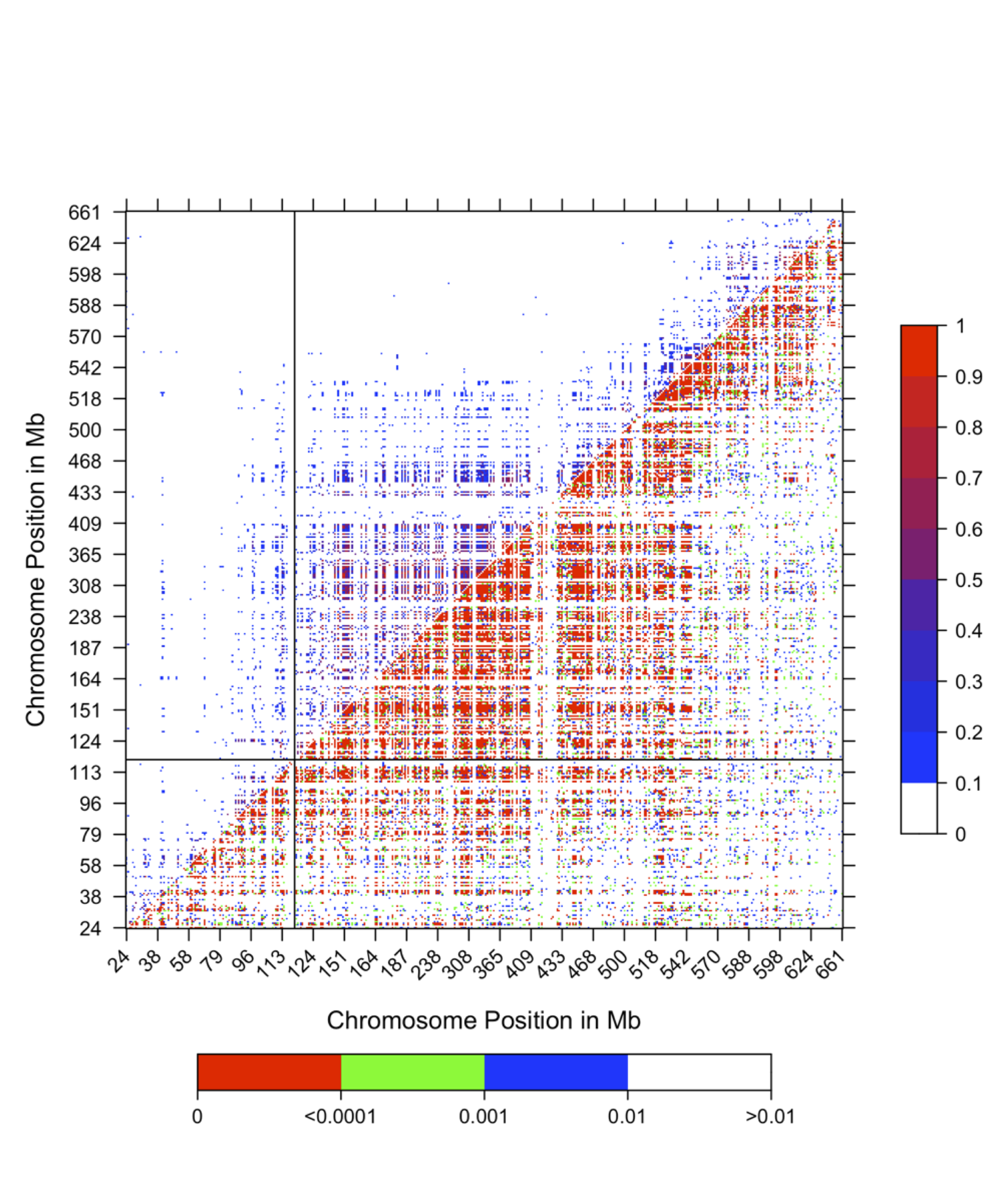

Supplement: Supplementary file 7 — Supplementary file7 Figure S7. Heat map of linkage disequilibrium (LD) across chromosome 3J with 461 single nucleotide polymorphic markers. Upper triangle is R2 values colored according to the key on the right, with the lower triangle showing p-values colored according to scale below the x-axis (PNG 2184 KB) [file 122_2022_4148_MOESM7_ESM.png]
