# Supplementary material for: Genetic architecture and QTL selection response for Kernza perennial grain domestication traits
Source: Theor Appl Genet. 2022 Jun 28;135(8):2769–84. doi: 10.1007/s00122-022-04148-2 (PMC9243872; doi:10.1007/s00122-022-04148-2)
Supplement: Supplementary file 8 — Supplementary file8 Table S1 Descriptive statistics, including total number and number of genets by cycle, of the best linear unbiased predictors of 34 traits measured in The Land Institute intermediate wheatgrass breeding program cycles 6-9 during 2016-2020 (DOCX 19 KB) [file 122_2022_4148_MOESM8_ESM.docx]

**Supporting Information Table S1** Descriptive statistics, including total number and number of genets by cycle, of the best linear unbiased predictors of 34 traits measured in The Land Institute intermediate wheatgrass breeding program cycles 6-9 during 2016-2020

| **Trait** | **C6 n** | **C7 n** | **C8 n** | **C9 n** | **Total n** | **Min.** | **Max.** | **Mean** | **STD** |
| --- | --- | --- | --- | --- | --- | --- | --- | --- | --- |
| Brittle Rachis | 0 | 1186 | 947 | 955 | 3088 | 0.49 | 1.63 | 0.99 | 0.17 |
| Seed Image Circularity | 1013 | 1151 | 940 | 926 | 4030 | 0.37 | 0.55 | 0.45 | 0.02 |
| Spike Emergence (cm) | 0 | 1180 | 0 | 0 | 1180 | 1.8 | 36.69 | 19.79 | 6.13 |
| Seed Length (mm) | 1013 | 1183 | 976 | 926 | 4098 | 5.07 | 8.13 | 6.68 | 0.41 |
| Seed Width (mm) | 1013 | 1183 | 976 | 926 | 4098 | 1.29 | 1.8 | 1.55 | 0.07 |
| Flag Leaf Length (cm) | 982 | 0 | 0 | 0 | 982 | 20.18 | 36.03 | 27.87 | 2.23 |
| Flag Leaf Width (mm) | 982 | 0 | 0 | 0 | 982 | 8.83 | 23.79 | 15.75 | 1.91 |
| Flag Leaf Height (cm) | 924 | 0 | 0 | 0 | 924 | 37.51 | 78.12 | 63.86 | 5.71 |
| Number of Florets per Spike | 1019 | 1176 | 873 | 0 | 3068 | 123.17 | 230.38 | 174.13 | 14.49 |
| Number of Florets per Spikelet | 1019 | 1176 | 873 | 0 | 3068 | 6.41 | 9.79 | 7.66 | 0.44 |
| Spike Emergence % | 918 | 1130 | 0 | 0 | 2048 | 0.05 | 1.31 | 0.64 | 0.18 |
| Lodging | 0 | 1182 | 938 | 0 | 2120 | 2.42 | 8.89 | 5.98 | 1.05 |
| Free Threshing | 1014 | 1184 | 979 | 937 | 4114 | 2.3 | 98.37 | 57.17 | 17.1 |
| Peduncle Width (mm) | 0 | 1150 | 0 | 0 | 1150 | 7.71 | 11.99 | 9.9 | 0.69 |
| Floret Site Utilization | 1012 | 1173 | 864 | 0 | 3049 | 0.12 | 0.39 | 0.22 | 0.03 |
| Plant Height (cm) | 1020 | 1191 | 992 | 954 | 4157 | 82.87 | 136.54 | 111.03 | 5.92 |
| Seed Area (mm2) | 1013 | 1183 | 976 | 926 | 4098 | 5.39 | 10.59 | 8.08 | 0.68 |
| Seed Density | 1013 | 1183 | 976 | 926 | 4098 | 0.97 | 1.67 | 1.31 | 0.08 |
| Seeds per Spike | 1016 | 1186 | 980 | 958 | 4140 | 10.7 | 62.24 | 38.21 | 6.8 |
| Seed Mass (mg) | 1013 | 1183 | 977 | 937 | 4110 | 5.89 | 13.34 | 9.46 | 1.08 |
| Seed Perimeter (mm) | 1013 | 0 | 0 | 0 | 1013 | 11.2 | 17.55 | 14.73 | 0.95 |
| Shattering | 1019 | 1188 | 981 | 948 | 4136 | 0.17 | 3.79 | 2.13 | 0.57 |
| Spikelet Density | 1017 | 0 | 0 | 0 | 1017 | 0.52 | 1.06 | 0.72 | 0.06 |
| Spike Dry Weight (g) | 0 | 1167 | 955 | 959 | 3081 | 0.83 | 1.75 | 1.31 | 0.13 |
| Spiklets per Spike | 1019 | 1176 | 873 | 0 | 3068 | 18.02 | 27.36 | 22.69 | 1.13 |
| Spike Harvest Index | 0 | 1156 | 955 | 958 | 3069 | 0.11 | 0.46 | 0.3 | 0.05 |
| Spike Length (cm) | 1017 | 1141 | 0 | 0 | 2158 | 22.59 | 45.88 | 34.78 | 2.86 |
| Spike Yield (g) | 1019 | 1188 | 981 | 958 | 4146 | 0.14 | 0.58 | 0.36 | 0.06 |
| Stem Angle | 1022 | 1179 | 0 | 0 | 2201 | 19.26 | 82.33 | 53.86 | 9.99 |
| Stem Strength Bottom | 914 | 0 | 0 | 0 | 914 | 1214.48 | 3424.35 | 2181.99 | 344.09 |
| Stem Diameter (mm) | 946 | 0 | 0 | 0 | 946 | 2.24 | 3.89 | 3.01 | 0.21 |
| Stem Strength Middle | 915 | 1174 | 0 | 0 | 2089 | 747.15 | 1910.92 | 1197.3 | 166.77 |
| Stem Strength Top | 915 | 0 | 0 | 0 | 915 | 288.65 | 615.32 | 418.66 | 53.05 |
| Maturity | 1017 | 1189 | 944 | 953 | 4103 | 52.21 | 69.9 | 63.14 | 1.48 |

**Supporting Information Table S2** Significant marker trait associations for The Land Institute intermediate wheatgrass breeding program analyzed by combined data and cycle-year combinations

QTL#, number of QTL; Associated markers, number of markers associated with each QTL; Chr, chromosome; n, number of individuals observed; *F_ST_* fixation index between The Land Institute (TLI) Cycle 6 and TLI Cycle 9; PVE, percent variance explained; Ref/Alt reference and alternate allele respectively; MAF

**Supporting Information Table S3** Chromosome location of genome-wide associations by trait for combined analysis (C) and individual cycle combinations (6-9) for The Land Institute intermediate wheatgrass breeding program

| **Trait** | **Chromosome** | | | | | | | | | | | | | | | | |
| --- | --- | --- | --- | --- | --- | --- | --- | --- | --- | --- | --- | --- | --- | --- | --- | --- | --- |
|  | **2J** | **3J** | **4J** | **5J** | **6J** | **7J** | **1S** | **2S** | **3S** | **4S** | **5S** | **7S** | **1V** | **2V** | **3V** | **5V** | **6V** |
| Brittle rachis |  | C,7,8,9 |  |  |  |  |  |  | C,7,9 |  | C,7 | 9 | 7 |  |  | 7 |  |
| Flag leaf length |  | 6 |  |  |  |  |  |  |  |  |  |  |  |  |  |  |  |
| Flag leaf width |  |  |  |  |  |  |  |  |  |  |  |  | 6 |  |  | 6 |  |
| Floret site utilization |  |  |  |  |  |  |  |  |  |  | C,7 |  |  |  |  |  |  |
| Free threshing |  |  |  |  |  |  |  |  |  |  |  |  |  | C,9 |  |  |  |
| Maturity | 7 |  |  |  | 6 |  |  |  |  |  |  |  |  |  |  |  |  |
| Number of florets per spike |  |  |  | C |  |  |  |  |  |  |  |  |  |  | C |  |  |
| Number of florets per spikelet | C |  |  | C |  |  |  |  |  |  |  |  |  |  |  |  |  |
| Peduncle width | C,7 |  |  |  |  |  |  |  |  |  |  |  |  |  |  |  |  |
| Plant height |  |  |  |  | 7,8 |  |  |  |  |  |  |  |  |  |  |  |  |
| Seed area |  |  |  |  |  | 6 |  |  |  |  |  | 8 |  |  |  |  |  |
| Seed density |  |  |  |  |  |  | C |  |  |  |  | C |  |  |  |  |  |
| Seed image circularity |  | C |  |  |  |  | C,7,8 |  |  | C,6 |  | C,6,7,8 | C,6,7 | C |  | C,6,7 |  |
| Seed length |  |  |  |  |  |  |  |  |  |  |  | 7 | C |  |  |  | C |
| Seed width | C |  | C,7 |  |  | 9 |  |  |  |  |  |  |  | C,6 |  |  |  |
| Shattering | C |  |  |  |  |  | 7 | C,7,8,9 | 7 | C,7,8,9 | C,7,8 |  |  | 7 |  |  |  |
| Spike emergence | C,7 |  |  |  |  |  |  |  |  |  |  |  |  |  |  |  |  |
| Spike emergence % | C |  |  |  |  |  |  |  |  |  |  |  |  |  |  |  |  |
| Spike length |  |  |  |  |  |  |  |  |  |  | C |  | 7 |  |  |  |  |
| Spikelet density |  |  |  |  |  |  |  |  | 6 |  |  |  |  |  | 6 |  |  |
| Spikelets per spike |  |  |  |  |  |  |  | C |  |  |  | 6 |  |  |  |  |  |
| Stem strength bottom |  |  |  |  |  |  |  |  | 6 |  |  |  |  |  |  |  |  |
| Stem diameter |  |  |  |  |  | C,6 |  |  |  |  |  |  |  |  |  |  |  |
| Stem strength middle |  |  |  |  |  |  |  |  | 6 |  |  |  |  |  |  |  |  |
