# Supplementary material for: Genetic architecture and QTL selection response for Kernza perennial grain domestication traits
Source: Theor Appl Genet. 2022 Jun 28;135(8):2769–84. doi: 10.1007/s00122-022-04148-2 (PMC9243872; doi:10.1007/s00122-022-04148-2)
Supplement: Supplementary file 10 — Supplementary file10 Table S3 Chromosome location of genome-wide associations by trait for combined analysis (C) and individual cycle combinations (6-9) for The Land Institute intermediate wheatgrass breeding program (DOCX 20 KB) [file 122_2022_4148_MOESM10_ESM.docx]

**Supporting Information Table S3** Chromosome location of genome-wide associations by trait for combined analysis (C) and individual cycle combinations (6-9) for The Land Institute intermediate wheatgrass breeding program

| **Trait** | **Chromosome** | | | | | | | | | | | | | | | | |
| --- | --- | --- | --- | --- | --- | --- | --- | --- | --- | --- | --- | --- | --- | --- | --- | --- | --- |
|  | **2J** | **3J** | **4J** | **5J** | **6J** | **7J** | **1S** | **2S** | **3S** | **4S** | **5S** | **7S** | **1V** | **2V** | **3V** | **5V** | **6V** |
| Brittle rachis |  | C,7,8,9 |  |  |  |  |  |  | C,7,9 |  | C,7 | 9 | 7 |  |  | 7 |  |
| Flag leaf length |  | 6 |  |  |  |  |  |  |  |  |  |  |  |  |  |  |  |
| Flag leaf width |  |  |  |  |  |  |  |  |  |  |  |  | 6 |  |  | 6 |  |
| Floret site utilization |  |  |  |  |  |  |  |  |  |  | C,7 |  |  |  |  |  |  |
| Free threshing |  |  |  |  |  |  |  |  |  |  |  |  |  | C,9 |  |  |  |
| Maturity | 7 |  |  |  | 6 |  |  |  |  |  |  |  |  |  |  |  |  |
| Number of florets per spike |  |  |  | C |  |  |  |  |  |  |  |  |  |  | C |  |  |
| Number of florets per spikelet | C |  |  | C |  |  |  |  |  |  |  |  |  |  |  |  |  |
| Peduncle width | C,7 |  |  |  |  |  |  |  |  |  |  |  |  |  |  |  |  |
| Plant height |  |  |  |  | 7,8 |  |  |  |  |  |  |  |  |  |  |  |  |
| Seed area |  |  |  |  |  | 6 |  |  |  |  |  | 8 |  |  |  |  |  |
| Seed density |  |  |  |  |  |  | C |  |  |  |  | C |  |  |  |  |  |
| Seed image circularity |  | C |  |  |  |  | C,7,8 |  |  | C,6 |  | C,6,7,8 | C,6,7 | C |  | C,6,7 |  |
| Seed length |  |  |  |  |  |  |  |  |  |  |  | 7 | C |  |  |  | C |
| Seed width | C |  | C,7 |  |  | 9 |  |  |  |  |  |  |  | C,6 |  |  |  |
| Shattering | C |  |  |  |  |  | 7 | C,7,8,9 | 7 | C,7,8,9 | C,7,8 |  |  | 7 |  |  |  |
| Spike emergence | C,7 |  |  |  |  |  |  |  |  |  |  |  |  |  |  |  |  |
| Spike emergence % | C |  |  |  |  |  |  |  |  |  |  |  |  |  |  |  |  |
| Spike length |  |  |  |  |  |  |  |  |  |  | C |  | 7 |  |  |  |  |
| Spikelet density |  |  |  |  |  |  |  |  | 6 |  |  |  |  |  | 6 |  |  |
| Spikelets per spike |  |  |  |  |  |  |  | C |  |  |  | 6 |  |  |  |  |  |
| Stem strength bottom |  |  |  |  |  |  |  |  | 6 |  |  |  |  |  |  |  |  |
| Stem diameter |  |  |  |  |  | C,6 |  |  |  |  |  |  |  |  |  |  |  |
| Stem strength middle |  |  |  |  |  |  |  |  | 6 |  |  |  |  |  |  |  |  |
